# Accounting for careless and insufficient effort responding in large-scale survey data—development, evaluation, and application of a screen-time-based weighting procedure

**DOI:** 10.3758/s13428-022-02053-6

**Published:** 2023-03-03

**Authors:** Esther Ulitzsch, Hyo Jeong Shin, Oliver Lüdtke

**Affiliations:** 1 IPN—Leibniz Institute for Science and Mathematics Education, Educational Measurement, Olshausenstraße 62, 24118 Kiel, Germany; 2 Centre for International Student Assessment, Munich, Germany; 3 Sogang University, Seoul, South Korea

**Keywords:** Careless responding, Screen times, Data screening, Item response theory, Maximum pseudo-likelihood estimation, Finite mixture modeling

## Abstract

Careless and insufficient effort responding (C/IER) poses a major threat to the quality of large-scale survey data. Traditional indicator-based procedures for its detection are limited in that they are only sensitive to specific types of C/IER behavior, such as straight lining or rapid responding, rely on arbitrary threshold settings, and do not allow taking the uncertainty of C/IER classification into account. Overcoming these limitations, we develop a two-step screen-time-based weighting procedure for computer-administered surveys. The procedure allows considering the uncertainty in C/IER identification, is agnostic towards the specific types of C/IE response patterns, and can feasibly be integrated with common analysis workflows for large-scale survey data. In Step 1, we draw on mixture modeling to identify subcomponents of log screen time distributions presumably stemming from C/IER. In Step 2, the analysis model of choice is applied to item response data, with respondents’ posterior class probabilities being employed to downweigh response patterns according to their probability of stemming from C/IER. We illustrate the approach on a sample of more than 400,000 respondents being administered 48 scales of the PISA 2018 background questionnaire. We gather supporting validity evidence by investigating relationships between C/IER proportions and screen characteristics that entail higher cognitive burden, such as screen position and text length, relating identified C/IER proportions to other indicators of C/IER as well as by investigating rank-order consistency in C/IER behavior across screens. Finally, in a re-analysis of the PISA 2018 background questionnaire data, we investigate the impact of the C/IER adjustments on country-level comparisons.

Large-scale surveys pose a rich and important source of data in psychology, social, and educational sciences. Commonly, large-scale surveys comprise self-reports on personality, attitude, interests, beliefs, and behavior from large, representative samples. The analysis of such data rests on the assumption that differences in item responses reflect differences in the constructs to be measured. Careless and insufficient effort responding (C/IER) poses a major threat to this assumption and, as such, to the quality and validity of conclusions drawn from large-scale survey data. C/IER occurs when respondents do not invest effort into carefully reading the survey instructions or the item content and selecting relevant response options. Instead of resulting in responses that are reflective of the constructs to be measured, C/IER may manifest itself in a plethora of behavioral patterns, ranging from random responding, through marking distinct patterns such as straight or diagonal lines, to providing no response at all. Left unconsidered, C/IER may distort associations among constructs of interest as well as psychometric properties such as reliability and factor structure (Huang, Curran, Keeney, Poposki, & DeShon, 2012; Woods, 2006; Schmitt & Stuits, 1985; DeSimone, DeSimone, Harms, & Wood, 2018; Kam & Meyer, 2015).

A case in point are the background questionnaires (BQs) administered in educational large-scale assessments (LSAs) such as the Programme for the International Assessment of Adult Competencies (PIAAC; OECD, 2013), the National Assessment of Educational Progress (NAEP; National Center for Education Statistics, 2009) or the Programme for International Student Assessment (PISA; OECD, 2020). These measures provide a valuable source of information for investigating the relationship between non-cognitive constructs and performance at the group level. LSA BQs are typically quite long (in PISA 2018, for instance, respondents received over 300 items). Due to the cognitive burden imposed by questionnaire length (see Bradburn, 1978) in combination with BQs’ low-stakes nature, respondents may be unable and/or unwilling to invest effort into responding to all items administered, thus lowering data quality.

A common approach to take C/IER into account is to exclude respondents—or, in most recent developments, single responses (Kroehne, Buchholz, & Goldhammer, 2019)—based on indicators that scan response patterns and/or collateral information such as timing data for aberrances presumably stemming from C/IER (see Curran, 2016; Meade & Craig, 2012; Niessen, Meijer, & Tendeiro, 2016; Huang et al., 2012, for overviews). The approach, however, is limited in that it heavily relies on somewhat arbitrary decisions on threshold settings on normative response patterns and times, and in that the uncertainty in C/IER classification is not taken into account. More sophisticated weighting and model-based approaches overcome these limitations. However, these approaches are currently either capable of detecting only some specific types of C/IE response patterns (Hong & Cheng, 2019), or are computationally too demanding to pose feasible alternatives in the context of large-scale data sets (Ulitzsch et al., 2021; Ulitzsch, Yildirim-Erbasli, Gorgun, & Bulut, 2022).

The aim of the present study is to develop, validate, and illustrate an intermediate approach that comprises the advantages of taking C/IER identification uncertainty into account on the one hand and possessing a light computational footprint and ease and flexibility of implementation on the other. To this end, we draw on the additional information on response behavior contained in timing data retrieved from computerized questionnaire administrations. In what follows, we first discuss the utility and applicability of current approaches to adjust for C/IER in large-scale data sets. We then present a stepwise weighting procedure that draws on screen time information to determine the probability that a respondent engaged in C/IER and then downweighs responses presumably stemming from C/IER in the analysis of response data. As we see the analysis of LSA BQ data as a major use case for this procedure, we illustrate it on data from the PISA 2018 (OECD, 2020) BQ, closely mirroring operational practice. We gather first supporting validity evidence for the proposed procedure by relating identified C/IER prevalences and trajectories to common behavioral indicators of C/IER as well as previous findings and theoretical considerations on its occurrence. Finally, we investigate whether the adjustment procedure alters conclusions on the PISA 2018 BQ constructs, and discuss its potential for the analysis of large-scale survey data.

## Approaches for careless and insufficient effort responding

In applied settings, C/IER detection has predominately relied on indicator-based approaches (e.g., Meade & Craig, 2012), scanning response patterns and/or timing data for aberrances potentially stemming for C/IER. Only recently, psychometric response-pattern-based weighting (Hong & Cheng, 2019) and model-based mixture modeling approaches (e.g., Ulitzsch et al., 2021; van Laar & Braeken, 2022) have been developed. Each class of approaches possesses its own unique set of assumptions and methods for distinguishing between attentive responding and C/IER and comes with different advantages and limitations. In the following, these will shortly be reviewed.

### Indicator-based approaches

#### Response-pattern-based indicators

Traditional approaches for C/IER commonly employ response-pattern-based indicators for its identification (see Niessen et al., 2016; Curran, 2016; Meade & Craig, 2012, for overviews) and exclude respondents exceeding a pre-defined threshold from further analyses. Examples for such indicators are the long string index as an indicator of response invariability, being constructed by examining the longest sequence of subsequently occurring identical responses for each respondent (Johnson, 2005), or the even-odd index as an indicator of response (in)consistency, given by the within-person correlation between the responses to odd-numbered and even-numbered items belonging to the same scale, averaged across scales (Curran, 2016; Huang et al., 2012). In a similar vein, person-fit statistics can be employed to identify response patterns that are unlikely to be observed given an assumed statistical model for item responses. The $l^{*}_{z}$ statistic (developed by Drasgow, Levine, & Williams, 1985, employed for C/IER classification by Niessen et al., 2016, and Patton, Cheng, Hong, & Diao, 2019), for instance, quantifies the likelihood of observing a response vector under a given item response theory (IRT) model. In the case that the $l^{*}_{z}$ statistic is very low, the response pattern strongly deviates from what could be expected based on the employed IRT model, and the pattern is classified as aberrant.

##### Strengths and limitations of response-pattern-based indicators

Response-pattern-based indicators are widely applied in practice. They can easily be employed for cleaning data from paper-and-pencil-based and computer-administered questionnaires alike. A major limitation of response-pattern-based indicators, however, is that each indicator is tailored to the detection of a specific type of C/IER behavior but may be insensitive to others. The long string index, for instance, is well suited for detecting straight lining but does not detect diagonal lining or random responding. Conversely, the even-odd index is insensitive to straight lining since this results in consistent response patterns (Curran, 2016). Person-fit statistics have predominantly been evaluated for identifying uniform random responses (Niessen et al., 2016; Patton et al., 2019). As a remedy to this issue, Curran (2016) suggested a sequential multiple-hurdle approach that combines multiple indicators and filters out respondents with the most extreme values on each indicator. Up to date, however, there are no guidelines for implementing multiple-hurdle approaches.

#### Timing-based indicators

Another alternative is to capitalize on information contained in other types of behavioral information besides item responses. With the widespread employment of computerized questionnaire administrations, indicators based on screen times or, if available, item-level response times have received increasing attention. Due to the absence of cognitive processing required for attentively evaluating the item, retrieving relevant information, and selecting a relevant response, aberrantly short response times or times spent on screen can be seen as indicators of C/IER. Thresholds used for distinguishing C/IER from attentive responses may be defined either based on an educated guess on the minimum amount of time required for an attentive response (e.g., 2 s per item, Huang et al., 2012; for investigations of the construct validity of this threshold see Bowling, Huang, Brower, & Bragg, 2021) or using visual inspection of the screen time or response time distribution (Kroehne et al., 2019; Wise, 2017).

Neighboring research on distinguishing rapid guessing from solution behavior in cognitive assessments offers a plethora of more advanced threshold setting techniques. These make use of the fact that in cognitive assessments items are commonly presented on separate screens, thus supporting to record item-level response times and identification of rapid guessing on the item-by-person level. For instance, based on the consideration that response time distributions comprised of both rapid guessing and solution behavior should be separable, mixture modeling can be employed for decomposing response times into multiple subcomponents. When only two mixture components are allowed, the lowest point between the two modes of the distribution can be located and set as the threshold for classifying respondents (Rios & Guo, 2020). Other methodological advances within this rich stream of research combine response accuracy with response times to inform threshold settings. These build on the assumption that the probability of a correct response should be higher for responses tracing back to solution behavior than for rapid guesses. For instance, thresholds may be set by considering proportions of correct responses conditional on response times and identifying the response time below which the proportion of correct responses is not higher than the chance level of success (Wise, 2017; Guo et al., 2016; Lee & Jia, 2014; see Nagy & Ulitzsch, 2021; Pokropek, 2016, for more elaborate model-based approaches). In a similar vein, other approaches (e.g., Wise, 2019; Frey, Spoden, Goldhammer, & Wenzel, 2018) leverage item-total correlations or (provisional) proficiency estimates to inform response time thresholds distinguishing between different types of test-taking behavior. Nevertheless, due to conceptual differences between rapid guessing in the context of cognitive assessments and C/IER in the context of questionnaires, borrowing from this broad array of methods developed for identifying rapid guessing for improving the detection of C/IER is not straightforward. Advanced methods for identifying rapid guessing in cognitive assessment are concerned with and leverage information on conditional proportions of correct responses. In non-cognitive assessments, however, there are no correct responses, and it is not self-evident how to derive hypotheses on differences in category probabilities or (provisional) estimates of non-cognitive traits between attentive and inattentive respondents to inform the setting of thresholds. Hence, approaches originating in research on rapid guessing need to be adapted with caution to the context of C/IER in non-cognitive assessment. Recently, Soland, Wise, and Gao (2019) provided an example of how this can be achieved. Mirroring approaches for rapid guessing that enrich response times with information on response accuracy, Soland et al., (2019) suggested inspecting response-pattern-based indicators for C/IER conditioned on median time per item derived from screen times, and identifying the time below which multiple of these indicators markedly differ from those for the entire sample. Note that the approach by Soland et al., (2019) identifies C/IER at the screen-by-respondent level.

##### Strengths and limitations of timing-based indicators

A major advantage of timing-based over response-pattern-based indicators is that these do not entail presumptions on the specific C/IE response patterns (i.e., random responding or straight lining) and, therefore, provide a more general measure of C/IER than response-pattern-based indicators. In support of this, multiple studies (e.g., Bowling et al., 2021; Huang et al., 2012; Huang, Liu, & Bowling, 2015; Soland et al., 2019) found high agreement between timing-based with various response-pattern-based indicators, each being sensitive to a different aspect of C/IER, as well as with other behavioral measures of C/IER, such as the inability to recognize item content. Nevertheless, this agnosticism concerning C/IE response patterns comes at the price of strong assumptions on times associated with C/IER and attentive responding, assuming the former to result in times that are always shorter than those associated with the latter.

A further advantage of timing-based over response-pattern-based indicators is that these potentially allow to identify C/IER on the item-by-respondent level, supporting finer-grained identification and bypassing discarding valuable information from respondents who engaged in C/IER only on some items. Note, however, that commonly multiple items are displayed on a single screen and timing data is, therefore, typically available only on the screen level. While item-level response times may be reconstructed, this requires the availability of additional process data and sophisticated data processing (Kroehne & Goldhammer, 2018).

#### Shared strengths and limitations of indicator-based approaches

Indicator-based approaches are widely applied in practice to investigate and adjust for C/IER (e.g., Magraw-Mickelson, Wang, & Gollwitzer, 2020; Huang et al., 2015; Nichols & Edlund, 2020; McKay, Garcia, Clapper, & Shultz, 2018; Bowling et al., 2016; Galesic & Bosnjak, 2009). Their major appeal lies in their straightforward implementation that can easily be integrated with standard data pre-processing procedures. Research aimed at validating whether C/IER indicators are capable of detecting C/IER commonly induced C/IER through varying instructions, e.g., by asking respondents to either proceed attentively or as quickly as possible through the questionnaire (e.g., Niessen et al., 2016; Bowling et al., 2021). In such studies, C/IER indicators have repeatedly been shown to be capable of detecting differences between these different groups of respondents.

A major limitation of indicator-based approaches to C/IER, however, is that they require a clear-cut decision on whether or not a respondent or an item response is classified as careless and, therefore, removed from subsequent analysis. Distributions of the employed indicators for attentive and careless behavior, however, are likely to overlap. When all items are worded in the same direction, high scores on the long string index due to repeatedly selecting the upper response option, for instance, may either go back to careless straight-lining behavior or may stem from attentive respondents with high trait levels. Screen or response times that are long enough that respondents may have had the chance to read an item but still markedly deviate from the average amount of time respondents require to generate a response may stem from respondents carelessly rushing through the questionnaire. They may, however, also indicate that respondents were very sure of their answer, thus requiring only a very short amount of time to generate an attentive response (referred to as distance-difficulty effect; Kuncel & Fiske, 1974). Further, not only very short but also outrageously long times entail ambiguity as to whether they stem from C/IER due to respondents getting distracted and not focusing on the administered items (Meade & Craig, 2012; Yildirim-Erbasli & Bulut, 2021), or go back to time-consuming attentive response processes, e.g., when respondents are indecisive between competing response options or require a large amount of time for processing the item content. Requiring a clear-cut decision for such cases neglects the uncertainty in classification and inevitably results in misclassifications. Both types of misclassifications may pose a threat to validity of conclusions. While falsely classifying careless respondents as attentive leaves attentive responses confounded with C/IER, falsely classifying attentive respondents as careless discards valuable information contained in the falsely classified attentive responses, and, even more important, may result in systematically excluding specific subgroups of attentive respondents (e.g., those providing their responses faster than average).

### Response-pattern-based weighting approach

Hong and Cheng (2019) proposed a response-pattern-based weighting approach for C/IER that utilizes person-fit statistics to downweigh response patterns according to their implausibility given an assumed polytomous IRT model, such as the graded response model (GRM; Samejima, 2016) or the generalized partial credit model (GPCM; Muraki, 1997). In Step 1 of this sequential approach, an IRT model for polytomous data is fit to the responses, and item parameter and person trait estimates are retrieved. In Step 2, person-fit statistics under the estimated IRT model are computed. Hong and Cheng (2019) introduced the approach employing the $l^{*}_{z}$ statistic (Sinharay, 2016), but noted that the approach can be implemented with other person-fit measures as well. Finally, in Step 3, fitting the same IRT model as in Step 1 to the data, person-fit statistics are employed as weights to downweigh implausible response patterns presumably stemming from C/IER. Weights are constructed such that the less plausible a response pattern under the given IRT model, the lower its contribution to the likelihood function of the IRT model used in the main analyses. Hong and Cheng (2019) noted that their approach may also be extended to iteratively updating person-fit statistics and IRT parameter estimates until some convergence criterion is met (as in Patton et al., 2019).

#### Strengths and limitations of the response-pattern-based weighting approach

The response-pattern-based weighting approach by Hong and Cheng (2019) overcomes major limitations of previous indicator-based procedures in that it avoids the setting of thresholds and allows to consider the uncertainty of C/IER classification in analyses. Further, Hong and Cheng (2019) noted that their approach is also capable of handling partial carelessness because a response pattern entirely going back to C/IER can be expected to result in a smaller weight, whereas a response pattern comprising both attentive and careless responses should receive a higher weight. From a practical perspective, the response-pattern-based weighting approach by Hong and Cheng (2019) can easily be implemented in any IRT package that supports the inclusion of respondent weights.

Nevertheless, the C/IER adjustment of the response-pattern-based weighting approach is only sensitive to aberrances that are detectable by the person-fit statistic employed. As shown by Hong and Cheng (2019), the employed $l^{*}_{z}$ statistic can be expected to perform well for detecting uniform random responding and random responding around the midpoint. It may, however, fail to flag other types of C/IER as aberrant. Straight lining, for instance, may result in response patterns with high plausibility under the employed IRT model, e.g., when items are rather homogeneous and/or when straight lining occurs on the outer response categories. Such patterns would receive high weights, neglecting the plausibility that they may also go back to C/IER. A further limitation of the approach by Hong and Cheng (2019) is that it is only applicable to IRT-based analyses of item response data and relies on well-constructed scales that align with the employed IRT model. Otherwise, poor person fit may trace back to misspecifications of the IRT model rather than C/IER, resulting in biased C/IER adjustments.

Note that the approach by Hong and Cheng (2019) is a method that can solely be employed for purifying estimates of item and structural parameters from C/IER. It does not support investigating its occurrence, i.e., does not provide an estimate of C/IER prevalence in the data at hand.

### Model-based mixture approaches

Recent developments of model-based mixture approaches for C/IER translate theoretical considerations on response behavior into different component models associated with attentive responding and C/IER. Model-based mixture approaches differ in the level at which C/IER is identified (respondent, screen-by-respondent, or item-by-respondent level) as well as the assumptions they incorporate on behavior of attentive and C/IE respondents.

The factor mixture modeling approach to C/IER by Arias, Garrido, Jenaro, Martinez-Molina, and Arias (2020) distinguishes between C/IE and attentive respondents by assuming that different factor structures hold in each latent group (i.e., latent class; see also Steinmann, Strietholt, & Braeken, 2022, for a stricter variant of the factor mixture modeling approach). Their model states that correlations between attentive responses go back to the underlying trait to be measured, while correlations between C/IE responses are induced by respondents’ category preferences (i.e., straight-lining behavior, see also Woods, 2006). The model requires both positively and negatively worded items, that, depending on the latent class, either give rise to homogeneous loadings, or to positive and negative loadings.

The mixture IRT approach for detecting random responders by van Laar and Braeken (2022) assumes an IRT model for polytomous data to hold for attentive respondents, while C/IE respondents are assumed to provide uniform random responses. This assumption is reflected in constraining all C/IER response category probabilities to be equal and unrelated to the trait to be measured.

For the analysis of questionnaires measuring multiple traits, both Arias et al., (2020) and van Laar and Braeken (2022) suggested applying the model to each trait separately. Note that the approaches by Arias et al., (2020) and van Laar and Braeken (2022) identify C/IER on the person level, and are tailored to the detection of specific C/IER behaviors (i.e., straight lining, respectively, uniform random responding).

These limitations are overcome in recently developed latent-response mixture modeling approaches (Ulitzsch et al., 2021; Ulitzsch et al., 2022). These allow for C/IER to vary at the screen-by-respondent or item-by-respondent level and avoid assumptions on specific C/IE response patterns. To facilitate distinguishing between C/IE and attentive responses, latent-response models leverage collateral item-level information, either drawing on response times from computerized questionnaires (Ulitzsch et al., 2021) or using information on item features such as position or text length (Ulitzsch et al., 2022). For attentive responses and, if considered, response times, customary models for polytomous data (e.g., Muraki, 1997) and response times from non-cognitive assessments (Ferrando & Lorenzo-Seva, 2007; Molenaar, Tuerlinckx, Maas, & van der Maas, 2015) are employed. C/IE responses are modeled in terms of freely estimated marginal category probabilities over all types of C/IE response patterns, thereby accommodating a wide array of C/IE response patterns, ranging from random responding to the marking of distinct patterns, such as straight or diagonal lines. C/IE response times are assumed to be unaffected by person and item characteristics and to generally be shorter than attentive response times.^1^

#### Strengths and limitations of model-based mixture approaches

Model-based mixture approaches pose sophisticated tools for identifying and modeling C/IER. Similar to the approach by Hong and Cheng (2019), by drawing on mixture modeling techniques, they avoid the setting of arbitrary thresholds and take the uncertainty of C/IER classification into account. Similar to timing-based indicator procedures to C/IER (Huang et al., 2012; Kroehne et al., 2019), latent-response mixture modeling approaches do not entail assumptions on the specific types of C/IE response patterns in the data. In contrast to timing-based indicator procedures, however, latent-response models that leverage this information take the possibly complex processes underlying response times from non-cognitive assessments into account and do allow for response time distributions associated with attentive and C/IER behavior to overlap. This entails potentially greater flexibility in C/IER identification, given that assumptions concerning the structures imposed on item responses and response times hold.

However, as the approach by Hong and Cheng (2019), model-based mixture approaches constrain researchers in their choice of analysis model, requiring to employ a factor or IRT model, and rely on well-constructed scales that align with the component model assumed for attentive responses. A further limitation of mixture modeling approaches in general and latent-response mixture modeling approaches in particular is that these are complex psychometric models, rendering their integration with standard operational practice challenging. For instance, for analyzing PISA BQ data, each scale is analyzed using a multigroup IRT model, with country as the grouping variable (see OECD, 2020). Even for a relatively simple model as in van Laar and Braeken (2022), which identifies C/IER on the person level and imposes constraints on C/IE response patterns, accounting for C/IER in operational practice would result in a complex multigroup mixture IRT model, with a separate mixture proportion to be estimated for each country. An additional limitation of the response-time-based latent-response model is that it requires response times at the item level. As pointed out above, these are oftentimes not available in non-cognitive assessment data.

## Study rationale

The objective of this study was twofold. First, we aimed to develop an easy-to-implement weighting approach to C/IER that takes the uncertainty in C/IER identification into account. To this end, we propose leveraging the rich information contained in timing data retrievable from computer-administered questionnaires. We see the conduct of sensitivity analyses, investigating the robustness of item parameter estimates and population-level inferences against presumed C/IER behavior, as the major use case of such an approach.

Second, we aimed to scrutinize the proposed approach using PISA 2018 BQ data as an exemplary application. To this end, we addressed multiple research objectives, setting out to explore which conclusions on C/IER occurrence the proposed approach can yield when applied to typical large-scale survey data (RO1), conducting initial validity checks (RO2), and investigating the effects of the approach’s C/IER adjustments (RO3). For addressing RO1, we re-analyzed PISA 2018 BQ data, while closely mimicking operational practice. For addressing RO2, we specified the following hypotheses concerning country-level and person-level trajectories of C/IER, considering evidence for these hypotheses as supporting validity evidence for the proposed approach.

- *H*1 Based on previous studies on scale and questionnaire characteristics associated with the occurrence of C/IER (Galesic & Bosnjak, 2009; Bowling, Gibson, Houpt, & Brower, 2020), we expected C/IER proportions to be higher on scales with characteristics that may impose additional cognitive burden on respondents. We, therefore, hypothesized positive relationships between the considered scales’ C/IER proportions with their position in the questionnaire, the number of items, average text length, and—as it may be more demanding to choose a suitable option when the number of options to choose from is higher—the number of response options.
- *H*2 Omissions on questionnaires and cognitive tests are a commonly employed behavioral indicator of respondent attentiveness (see Galesic & Bosnjak, 2009; Boe, May, & Boruch, 2002; Wise & Gao, 2017; Ulitzsch, von Davier, & Pohl, 2020). We, therefore, expected identified C/IER behavior to coincide with increased omission behavior.
- *H*3 The proposed approach and previously employed timing-based indicators (Wise & Ma, 2012; Huang et al., 2012) both aim to identify C/IER employing the same type of data. We, therefore, hypothesized agreement with more heuristic timing-based indicators (Wise & Ma, 2012; Huang et al., 2012).^2^
- *H*4 Based on the consideration that C/IER may reflect enduring inter-individual differences that generalize across time and research contexts (Bowling et al., 2016) and, for that matter, across jointly administered scales, we expected rank-order consistency in C/IER behavior across scales.

For addressing RO3, we compared results retrieved under C/IER adjustments against those from standard analyses that leave C/IER unconsidered. We expected differences between these analyses to be more pronounced for higher proportions of C/IER identified by the proposed approach.

## Proposed approach

Our approach is designed for computerized questionnaire administrations in which items for each trait are presented on a separate screen and times spent on screen are recorded. The basic idea is to draw on mixture modeling to decompose log screen time distributions into several subcomponents, out of which the subcomponent with the lowest mean is assumed to stem from C/IER. For this subcomponent, respondent posterior C/IER class probabilities are computed. Finally, the analysis model of choice is applied to item response data. When doing so, posterior C/IER class probabilities are employed to downweigh response patterns presumably stemming from C/IER.

As in the approaches by Hong and Cheng (2019), Arias et al., (2020), and van Laar and Braeken (2022), C/IER is identified on the person level and each scale is analyzed separately. In what follows, we describe how the proposed approach identifies and adjusts for C/IER in the analysis of data from a single scale. For the sake of simplicity, we assume that all items from the same scale are presented on a joint screen, and make use of item responses and respondents’ times spent on the screen administering the scale of interest.

### Decomposing log screen times

Log screen time distributions are assumed to potentially stem from *C* qualitatively different types of response processes, and the aim is to decompose the distribution of log screen times into its *C* subcomponents by means of Gaussian mixture models. That is, respondent *i*’s, *i* = 1,...,*N*, log screen time $\ln (t_{i})$ belonging to subcomponent *c*, *c* = 1,...,*C*, is assumed to stem from a normal distribution with mean *μ*_*c*_ and variance ${\sigma ^{2}_{c}}$,

1 $$ \ln(t_{i}|z_{i}=c) \sim \mathcal{N}(\mu_{c},{\sigma^{2}_{c}}), $$

with *z*_*i*_ ∈{1,…,*C*} denoting respondent *i*’s unobserved component membership. Hence, the marginal distribution of $\ln (t_{i})$ is

2 $$ \begin{array}{@{}rcl@{}} f(\ln(t_{i}))&=& \sum\limits_{c=1}^{C}\pi_{c} \frac{1}{\sigma_{c}\sqrt{2\pi}} \exp\left( -\frac{1}{2}\left( \frac{\ln(t_{i})-\mu_{c}}{\sigma_{c}}\right)^{\!2} \right)\\ &&\text{with}\quad 0\leq\pi_{c}\leq1 \text{ and } \sum\limits_{c=1}^{C}\pi_{c}=1, \end{array} $$

where *π*_*c*_ = *p*(*z*_*i*_ = *c*) denotes the mixture proportion for component *c* (refer to McLachlan & Peel, 2004, for an introduction to finite mixture models and further details on identifiability).

For decomposing the screen time distribution into its subcomponents, we suggest fitting multiple Gaussian mixture models to log screen times, varying the number of mixture components *C*, and to determine the number of components by means of model comparisons. These can be conducted using information criteria, such as the Bayesian information criterion (BIC). In the case that log screen times are decomposed into at least two components (i.e., *C* ≥ 2), the component with the lowest mean is assumed to comprise of screen times associated with C/IER and labeled accordingly, that is

3 $$ c^{\text{C/IER}} = \underset{c \in \{1, \ldots, C\}}{\arg \min} \mu_{c}.  $$

For decluttering notation, the C/IER label is moved to the superscript position. The mixture proportion associated with the lowest mean *π*^C/IER^ is assumed to give the respondent-level proportion of C/IER on the considered screen. The remaining components are assumed to stem from attentive response processes.

Conceptually, the screen time decomposition aims to identify a screen time outlier class that, on average, spent only a relatively short amount of time on the considered screen. As will be illustrated below, this does not restrict C/IER identification to extremely short screen times but may, in the case that the C/IER components’ variance is sufficiently large, also identify inattentive respondents with times that exceed typical screen times of the distributions attributed to attentive responding.

We refrain from constraining the number of mixture components to two, implying one attentive and one C/IER component, as we aim to extract the presumed C/IER screen time component while modeling attentive screen times as flexibly as possible. Note that the approach is not aimed at providing substantive interpretations for the identified possibly multiple attentive screen time components. In fact, attentive screen time subcomponents may not necessarily be substantively meaningful. Reasons as to why the approach may identify multiple attentive subcomponents are manifold. The attentive log screen time distribution, for instance, may deviate from normality. If that is the case, modeling attentive screen times in terms of multiple normal components poses a flexible way to accommodate such deviations. Likewise, attentive screen times themselves may go back to multiple distinct processes, such as different answering strategies.

Nevertheless, it should be pointed out that the employment of log screen time mixture decomposition to identify C/IER is not without assumptions. While the approach can be expected to be rather flexible in accommodating different distributions of attentive screen times, C/IER log screen times are assumed to be normal. Further, it relies on the strong assumption that there is one and only one C/IER component in the case that log screen times are decomposed into at least two components—both the absence of C/IER in the face of multiple attentive components as well as the presence of multiple C/IER subcomponents pose plausible violations of this assumption.

### Retrieving attentiveness weights

After decomposing log screen time distributions, posterior C/IER class probabilities $\pi _{i}^{\text {C/IER}}=p(z_{i}=\text {C/IER}|\ln (t_{i}))$ are determined for each respondent. From the posterior C/IER class probabilities, respondent attentiveness weights are constructed as $w_{i}^{\text {Att}}=1-\pi _{i}^{\text {C/IER}}$. Attentiveness weights are constructed such that the less likely screen times belong to the class with the lowest mean log screen time, the higher the attentiveness weights, and the more the associated response pattern contributes to the likelihood function of the model fitted to item responses. We point out that timing-based threshold methods for C/IER can be seen as a special case of the proposed weighting procedure, where $w_{i}^{\text {Att}}$ is set to 0 in the case that respondent *i*’s screen time is below some pre-defined threshold and to 1 otherwise. That is, in the former case, respondent *i*’s responses are left unconsidered in parameter estimation.

**Fig. 1:**
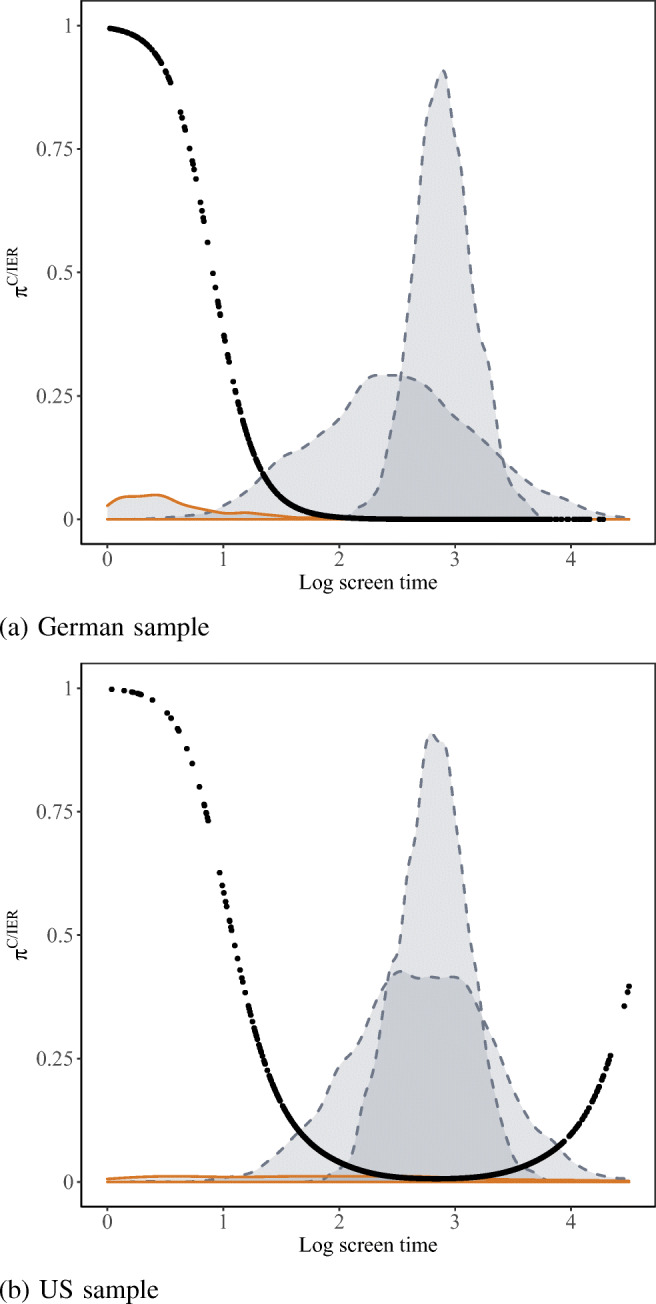
Decomposition of log time spent on screen ST206 of the PISA 2018 BQ. The presumed C/IER component is marked in *orange*, and the two attentive components are displayed in *blue-gray.* X-axes are truncated. Posterior C/IER class probabilities *π*^C/IER^ are superimposed

Schematic illustrations for Gaussian mixture models with three components are given in Fig. 1, displaying results for log time spent on screen ST206 from the German and US PISA 2018 BQ sample from the empirical application reported below. The presumed C/IER component is marked in orange, while the two attentive components are displayed in blue-gray. Posterior C/IER class probabilities are superimposed on the exemplary decomposed screen time distributions. As can be seen, extremely low screen times are attributed with high certainty to C/IER. Hence, response patterns associated with extremely low screen times receive attentiveness weights close to zero. Furthermore, as becomes evident from Fig. 1b, the proposed procedure may not only downweigh response patterns associated with very short screen times, but also those associated with screen times that exceed typical screen times of the distributions attributed to attentive responding. In the displayed US sample, for instance, some few respondents spent more than one minute (corresponding to a log screen time of 4.10) on the investigated four-item screen. For such respondents with aberrantly long screen times, posterior C/IER class probabilities reflect the uncertainty of determining whether these go back to C/IER (e.g., being distracted; see Yildirim-Erbasli & Bulut, 2021, for further discussions in the context of cognitive assessments) or attentive response processes that require more time than usual (e.g., being very indecisive or requiring a lot of time for attentively processing the item content).

### Considering attentiveness weights in parameter estimation

In analogy to the approach by Hong and Cheng (2019), attentiveness weights are employed to downweigh response vectors presumably stemming from C/IER when implementing the analysis model of choice. The rationale for doing so is that, conceptually, C/IER can be understood as a specific type of nonresponse that results in data that do not contain relevant information on parameters of interest.

Note that—like indicator-based approaches and in contrast to weighting and mixture model-based approaches—the proposed approach does not restrict analyses to specific model classes but provides researchers with great flexibility in employing analysis models of their choosing—ranging from simple subgroup mean sum scores to complex IRT models. We here illustrate how the proposed approach can be integrated with IRT-based analyses of item response data.

In the context of LSA BQs, the GPCM is a prominent choice for modeling item responses *y*_*ij*_ ∈{0…,*K*}, containing person *i*’s response to the *j* th item, *j* = 1,…,*J*, with *K* giving the highest possible response category for the considered items. Under the GPCM, the probability of respondent *i* to choose category *k*, *k* = 0,…,*K*, on the *j* th item is modeled as

4 $$ \begin{array}{@{}rcl@{}} p(y_{ij} &=& k| a_{j}, \boldsymbol{b}_{j}, \theta_{i}) = \frac{\exp({\sum}_{l=0}^{k} a_{j}(\theta_{i} - b_{jl}))}{{\sum}_{r=0}^{K}\exp({\sum}_{l=0}^{r} a_{j}(\theta_{i} - b_{jl}))}\\&& \text{with } \sum\limits_{l=0}^{0} a_{j}(\theta_{i} - b_{jl})\equiv0. \end{array} $$

Here, *𝜃*_*i*_ denotes respondent *i*’s trait level. The parameters *b*_*jl*_ and *a*_*j*_ give the *l* th step difficulty and discrimination of item *j*, respectively.

For parameter estimation, marginal maximum likelihood (MML) is typically employed. In MML estimation, latent variables *𝜃* are integrated out by posing some distributional assumption *F*_***γ***_ for *𝜃*, commonly choosing a normal distribution. MML seeks to maximize the log likelihood

5 $$ l(\boldsymbol{a},\boldsymbol{b},\boldsymbol{\gamma}) = \sum\limits_{i=1}^{N} \log p(\boldsymbol{y}_{i}|\boldsymbol{a},\boldsymbol{b},\boldsymbol{\gamma})  $$

with respect to the item parameters (***a***,***b***) and the distribution parameters ***γ***, e.g., the mean *μ*_*𝜃*_ and standard deviation *σ*_*𝜃*_ of the normal distribution assumed for *𝜃*. The likelihood contribution for respondent *i* is given by

6 $$ l_{i}(\boldsymbol{a},\boldsymbol{\!b},\boldsymbol{\gamma}) = \log \left[ \int \prod\limits_{j=1}^{J} p(y_{ij} = k| a_{j}, \boldsymbol{b}_{j}, \theta_{i}) dF_{\boldsymbol{\gamma}}(\theta)\right]. $$

For considering response vectors according to their probability of stemming from attentive responding, Eq. 5 can be modified to (see also Hong & Cheng, 2019)

7 $$ l(\boldsymbol{a},\boldsymbol{b},\boldsymbol{\gamma}) = \sum\limits_{i=1}^{N} w_{i}^{\text{Att}} \log p(\boldsymbol{y}_{i}|\boldsymbol{a},\boldsymbol{b},\boldsymbol{\gamma}).  $$

Note that such weighting of the likelihood function is similar to the so-called pseudo-likelihood approach for complex survey data (Thomas & Cyr, 2002). In fact, in the case that researchers want to consider possibly complex sampling designs by including sampling weights *v*_*i*_, these can simply be multiplied with the respondents’ attentiveness weights, that is

8 $$ l(\boldsymbol{a},\boldsymbol{b},\boldsymbol{\gamma}) = \sum\limits_{i=1}^{N} w_{i}^{\text{Att}}v_{i} \log p(\boldsymbol{y}_{i}|\boldsymbol{a},\boldsymbol{b},\boldsymbol{\gamma}).  $$

The OSF repository accompanying this article provides R code for decomposing screen time distributions and incorporating attentiveness weights in IRT analyses for a single scale and country-by-language group subsample from the PISA 2018 BQ data employed in the empirical application reported below.

## Empirical application

### Data

We based analyses on screen times and item responses from the computer-administered PISA 2018 BQ questionnaire (OECD, 2020).^3^ Due to the two-step language input methods required for entering a Chinese word and the associated increased cognitive demand and timely burden, timing data from the Chinese version of the BQ may not be comparable with those from non-Chinese language versions. We, therefore, excluded Chinese-speaking countries and economies (i.e., Beijing, Shanghai, Jiangsu, and Guangdong as well as Hong Kong, Macao, and Taipei). Further, we excluded countries with extensive customizations of the BQ, that is, countries that chose national adaptations for more than five of the BQ scales (i.e., Belgium, Canada, the Czech Republic, Denmark, Finland, Georgia, Israel, Japan, Luxembourg, the Netherlands, Norway, Qatar, Sweden, and the United States). To avoid screen time decompositions that reflect differences in language-specific time requirements rather than differences in response processes, we analyzed screen times separately for each country-by-language group. To ensure stability of screen time mixtures, we excluded country-by-language group with a sample size below 500. This resulted in a sample of 403,117 respondents from 68 country-by-language groups. Group-level sample size ranged from 529 (Russian language group in Lithuania) to 30,255 (Spanish language group in Spain).

We considered only scales that are reflective of non-cognitive constructs, thus scaled through IRT models. This led to the exclusion of screens concerning demographics, screens with open-ended questions or slider bars as well as single-item screens. An overview of the analyzed 48 scales, including selected scale characteristics, is given in Table 1. The first BQ scale considered was administered at screen position 21. The previous screens mainly collected information about demographic variables and administered instruction pages. Note that some scales were not administered in every country. The analyzed scales comprised a total of 234 items. 5.55% of item responses were missing due to item omissions.

**Table 1:** Overview over the analyzed PISA 2018 BQ scales

Scale	Construct	Screen position	Number of items	Number of options	Average length
ST097	Disciplinary climate	21	5	4	7.60
ST100	Teacher support	22	4	4	8.00
ST102	Teacher’s direct instruction	23	4	4	10.00
ST211	Perceived teacher support	24	3	4	11.00
ST212	Adaptation of instruction	25	3	4	14.67
ST104	Perceived feedback	26	3	4	10.67
ST213	Perceived teacher’s interest	27	4	4	9.75
ST150	Opportunity to learn in reading (material)	28	4	4	5.25
ST152	Teachers’ stimulation of reading and teaching strategies	29	4	4	12.00
ST153	Opportunity to learn in reading	31	9	2	11.67
ST158	Opportunity to learn digital reading	32	7	2	13.71
ST160	Reading engagement	33	5	4	8.80
ST167	Students’ reading practices (diverse reading material)	34	5	5	2.40
ST176	Students’ digital reading practices	37	6	5	7.33
ST161	Perceptions of competence/difficulty in reading	38	6	4	8.33
ST163	Perceptions of difficulty of the PISA test	39	3	4	9.00
ST164	Reading metacognition (understanding and remembering)	41	6	6	10.00
ST165	Reading metacognition (summarizing)	42	5	6	18.80
ST166	Digital reading metacognition (assess quality and credibility)	43	5	6	10.60
ST036	Value of school	45	3	4	9.67
ST181	Competitiveness	48	3	4	12.00
ST182	Work mastery	49	3	4	16.75
ST183	General fear of failure	50	3	4	14.00
ST185	Eudaemonia—meaning in life	52	3	4	10.00
ST186	Subjective well-being	53	9	4	1.00
ST208	Achievement goals	54	3	5	11.67
ST188	Resilience	55	5	4	11.00
ST034	Sense of belonging	56	6	4	8.00
ST196	Self-efficacy regarding global issues	57	6	4	10.50
ST197	Awareness of global issues	58	7	4	6.57
ST215	Perspective taking	59	5	5	16.40
ST216	Cognitive flexibility/adaptability	60	6	5	11.50
ST218	Awareness of intercultural communication	61	6	4	9.43
ST222	Engagement (with others) regarding global issues	62	8	2	14.13
ST214	Interest in learning about other cultures	63	4	5	11.25
ST220	Contact with people from other countries	64	4	2	3.25
ST217	Respect for people from other cultures	65	5	5	10.00
ST219	Global-mindedness	66	6	4	14.17
ST204	Attitudes towards equal rights for immigrants	67	4	4	15.75
ST177	Number of languages spoken	68	3	4	1.67
ST221	Learning activities (global issues)	70	10	2	12.80
ST223	Inclusive school climate	71	4	4	10.75
ST123	Student-parent relationship	72	3	4	8.67
ST205	Climate of competition	73	4	4	9.00
ST062	Loss in learning time (loss on individual level—truancy)	77	3	4	5.00
ST038	Bullying (own experience)	78	6	4	8.00
ST207	Bullying (attitude and beliefs)	79	5	4	11.00
ST206	Cooperation	80	4	4	9.25

Scales with two options comprise yes/no statements. Otherwise, scales comprise Likert-type items. Average length gives the average number of words per item in the English source version of the background questionnaire

To ease interpretation of C/IER time means, we based analyses on the log geometric mean time averaged across the number of items presented on the given screen. That is, the time *t*_*is*_ respondent *i* spent on screen *s*, $s=1,\dots , S$, was transformed as $\ln (t_{is}^{\frac {1}{J_{s}}})$, with *J*_*s*_ giving the number of items administered on screen *s*.

**Fig. 2:**
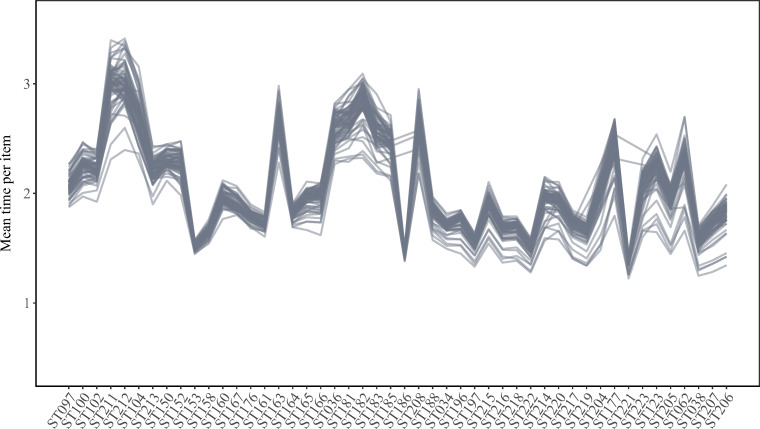
Country-by-language-group-level trajectories of average geometric mean time per item across the considered scales

Figure 2 gives country-by-language-level trajectories of geometric mean times per item across the considered scales. The average time per item was rather short (mean: 2.08 s, standard deviation: 0.65). Average time per item by country-by-language group and scale ranged from 1.22 s (Arabic language group in Morocco on scale ST221) to 3.41 s (Spanish language group in the Dominican Republic on scale ST212).

### Analyses

#### RO1: Identifying C/IER and investigating results

The analysis of screen times was implemented using the package mclust (Scrucca, Fop, Murphy, & Raftery, 2016), which allows for incorporating the PISA sampling weights. For each country-by-language group and scale, log geometric mean times per item were analyzed separately. A structure with one to nine mixture components was fit and the best solution with respect to the BIC was determined. Recall that in the proposed approach only solutions with at least two mixture components are assumed to indicate the occurrence of C/IER and that the component with the lowest mean is labeled as going back to C/IER (see Eq. 3).

To better understand the obtained results, we visually inspected a selected set of screen time decompositions. In these investigations, we aimed at better understanding results that stood out, and thus focused on group-by-scale combinations with unusually high C/IER proportions and spikes in C/IER proportions shared by multiple country-by-language groups.

#### RO2: Conducting initial validity checks

##### H1: Relating C/IER to scale characteristics

We analyzed the relationship between scale characteristics and the (presumed) occurrence of C/IER on the screen-by-group level using Beta regression, which is well suited for proportion data. To accommodate the nesting of scales within country-by-language groups and account for different baseline propensities to show C/IER across country-by-language groups, we ran a hierarchical random intercept model with the proportion of C/IER $\pi ^{\text {C/IER}}_{sg}$ on scale *s*, $s = 1,\dots ,S$, in country-by-language group *g*, $g = 1,\dots ,G$, (i.e., the mixture proportion for the C/IER component for each country-by-language-group screen time decomposition) being modeled as

9 $$ \begin{array}{@{}rcl@{}} \pi^{\text{C/IER}}_{sg} &\sim& \text{beta}(\mu_{sg}\phi,(1-\mu_{sg})\phi) \qquad \text{where } \\&&\mu_{sg} = \frac{\exp(\beta_{0g} + {\sum}_{p=1}^{P} \beta_{p} x_{ps})}{1+\exp(\beta_{0g} + {\sum}_{p=1}^{P} \beta_{p} x_{ps})}. \end{array} $$

The parameter *β*_0*g*_ gives the group-specific intercept, *β*_*p*_ denotes the fixed regression weight for the *p* th scale characteristic, $p=1,\dots ,P$, and *ϕ* is a precision parameter. Group-specific intercepts are assumed to be normally distributed with $\mathcal {N}(\mu _{\beta _{0}},\sigma _{\beta _{0}}^{2})$. Scale position, number of items, the average number of words per item in the English source BQ version as a proxy for text length, and the number of response options were considered as scale characteristics *x*_*ps*_. Bayesian estimation of the Beta regression was conducted using Stan version 2.19 (Carpenter et al., 2017) employing the rstan package version 2.19.3 (Guo, Gabry, & Goodrich, 2018). We ran two Markov chain Monte Carlo (MCMC) chains with 3,000 iterations each, with the first half being employed as warm-up. We employed diffuse normal priors with mean 0 and standard deviation 10 for the average intercept $\mu _{\beta _{0}}$ and the fixed regression weights *β*_*p*_. The standard deviation of the random intercept $\sigma _{\beta _{0}}$ and the precision parameter *ϕ* were equipped with half-Cauchy priors with location 0 and scale 5. The sampling procedure was assessed on the basis of potential scale reduction factor (PSRF) values, with PSRF values below 1.05 for all parameters being considered as satisfactory (Gelman & Rubin, 1992; Gelman & Shirley, 2011). We employed the posterior mean (EAP) as a Bayesian point estimate.

##### H2: Investigating agreement between item omissions and C/IER behavior

For investigating the relationship between item omissions and presumed C/IER behavior, we calculated country-by-language-group-level correlations between posterior C/IER class probabilities $\pi ^{\text {C/IER}}_{is}$ with the person-level number of item omissions for each scale, taking PISA sampling weights into account.

##### H3: Investigating agreement with timing-based indicators

For investigating agreement with timing-based indicators, we implemented three different threshold settings. For all threshold settings, C/IER was identified at the scale-by-respondent level, making use of the scale-level geometric mean time averaged across the given scale’s items. First, we implemented the customary 2-s threshold established by Huang et al., (2012), that is, constructed a C/IER indicator $d^{2s}_{is}$ storing information on whether or not person *i*’s interaction with scale *s* is classified as inattentive as

10 $$ d^{2s}_{is} = \begin{cases} 1 & \text{if } t_{is}^{\frac{1}{J_{s}}} < 2 \text{ s}\\ 0 & \text{otherwise}. \end{cases} $$

Note that the suitability of the 2-s threshold for the data at hand can be questioned, as it sets the threshold only 0.08 s below the average time per item. Therefore, second, to accommodate the short average time per item of 2.08 s in the investigated data set, we implemented a more conservative 1-s threshold, that is, constructed $d^{1s}_{is}$ as

11 $$ d^{1s}_{is} = \begin{cases} 1 & \text{if } t_{is}^{\frac{1}{J_{s}}} < 1 \text{ s}\\ 0 & \text{otherwise}. \end{cases} $$

Third, we borrowed from established threshold setting techniques originating in the context of cognitive assessments. We chose the normative threshold approach (Wise and Kuhfeld, 2021; Wise & Ma, 2012), as this approach makes use of timing data only (rendering it potentially applicable to the context of C/IER), is well validated (Wise & Ma, 2012), and implemented in operational settings (see Soland, Kuhfeld, & Rios, 2021). The normative threshold approach assumes inattentiveness to entail aberrantly short times, and sets thresholds to some low (but ultimately arbitrary) percentage (e.g., 10% or 30%) of an item’s mean time. We here implemented the 30% normative threshold approach (NT30) by constructing $d^{NT30}_{is}$ as

12 $$ d^{NT30}_{is} = \begin{cases} 1 & \text{if } t_{is}^{\frac{1}{J_{s}}} < 0.30 \frac{1}{N_{g[i]}} {\sum}_{i=1}^{N_{g[i]}} t_{is}^{\frac{1}{J_{s}}} \\ 0 & \text{otherwise}, \end{cases} $$

with *N*_*g*[*i*]_ giving the sample size of person *i*’s country-by-language group.

For investigating agreement with the proposed approach, we contrasted C/IER rates and trajectories implied by the different threshold settings against those given by the proposed approach, and calculated point-biserial correlations between timing-based indicators $d^{2s}_{is}$, $d^{1s}_{is}$, and $d^{NT30}_{is}$ and posterior C/IER class probabilities $\pi ^{\text {C/IER}}_{is}$.

##### H4: Investigating rank-order consistency in C/IER behavior

For investigating rank-order consistency in C/IER behavior across scales, we calculated country-by-language-group-level lagged correlations between posterior C/IER class probabilities for lags in scale positions from 1 to 15.

#### RO3: Investigating the impact of C/IER adjustments

For investigating the impact of C/IER adjustments, we contrasted the results of customary, unadjusted IRT analyses of the considered scales against those obtained from the proposed adjustment procedure. Following standard operational practice (OECD, 2020), each scale was analyzed separately using a multi-group GPCM, with country-by-language group as a grouping variable. Item parameters were assumed to be the same across groups. For identification, the latent mean and variance of the first group were set to 0 and 1, respectively. The remaining latent group means and variances were freely estimated. MML estimation of the multi-group models was implemented using the R package mirt version 1.35.1 (Chalmers, 2012), which supports the inclusion of respondent weights. In both analyses, we considered the PISA sampling weights. For the C/IER adjustments, sampling weights were multiplied with scale-by-person-specific attentiveness weights. We compared results in terms of country-by-language group means and variances of the analyzed scales.

All analyses were performed using R version 3.6.3 (R Development Core Team, 2017).

### Results

#### RO1: Identifying C/IER and investigating results

**Fig. 3:**
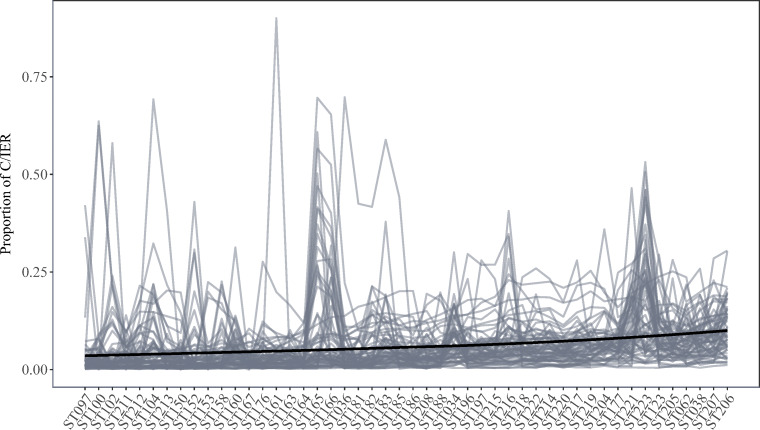
Country-by-language-group-level trajectories of C/IER proportions across the considered scales. A loess regression line is superimposed

Figure 3 displays country-by-language-group-level trajectories of C/IER proportions. Overall, average C/IER proportions oscillated around .06, with a slight increase with later scale positions. With grand means of .17 and .03, scales ST223 (administered at screen position 71) and ST167 (administered at screen position 34) exhibited the highest and lowest average C/IER proportion, respectively. For 14.96% of the scale-by-group combinations, C/IER proportions were below .01, indicating that the proposed approach may also detect infinitesimal C/IER prevalences. At the same time, only 0.15% of screen time decompositions yielded a one-class solution, i.e., the conclusion that respondents from the country-by-language group did not engage in C/IER on the screen. The majority of screen time decompositions yielded three (59.67%), four (22.47%), or two classes (11.05%). Screen time decompositions with two-class solutions yielded the highest average C/IER proportion of .16, followed by seven-class solutions (.08). Decompositions with four classes yielded the lowest average C/IER proportion of .04.

We observed systematic spikes for scale pairs ST165 and ST166 (C/IER grand means: .12 and .10) and ST221 and ST223 (C/IER grand means: .11 and .17). The former scales are scenario-based assessments of reading metacognition, asking students to rate strategies for summarizing a difficult text they received as a part of the Literacy assessment and for handling potential spam mail. It could be speculated that such scenario-based assessments are cognitively demanding, thus potentially eliciting higher levels of C/IER. The latter scale pair is part of the global competence assessment, and it remains unclear whether (and, if so, why) these scales may evoke higher levels of C/IER, or whether these spikes in presumed C/IER proportions are artifacts and go back to specific characteristics of the attentive screen time distribution resulting from differences in students’ cognitive processes when evaluating the items of these scales.

**Fig. 4:**
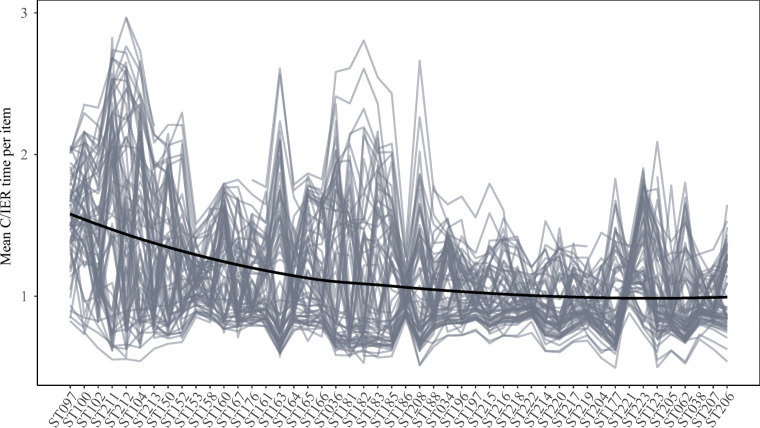
Country-by-language-group-level mean C/IER times per item across the considered scales. A loess regression line is superimposed

With an average mean C/IER time per item as low as 1.13 s, mean C/IER times (given in Fig. 4) remained far below the overall mean times per item (given in Fig. 2). Furthermore, mean C/IER time per item tended to decrease with later scale positions, which may indicate that respondents engaging in C/IER tended to accelerate with increasing proximity to the end of the questionnaire.

**Fig. 5:**
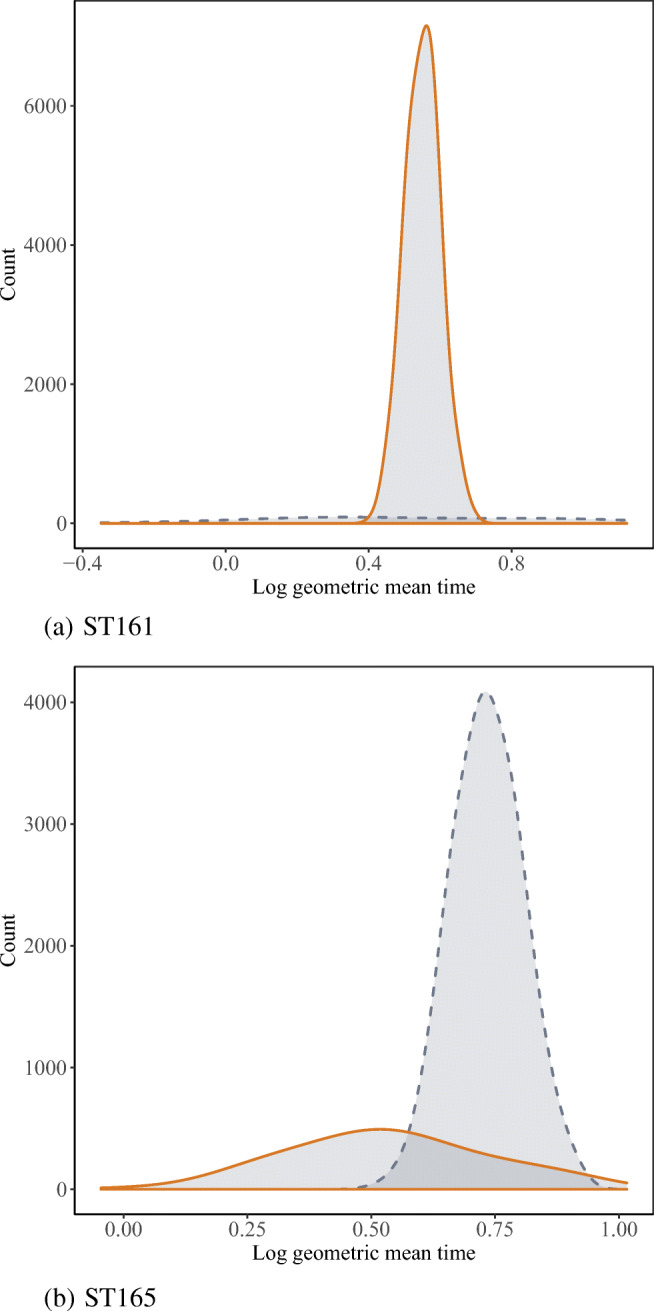
Decomposition of log geometric time spent on screens ST161 and ST165 for the Italian language group in Italy. The presumed C/IER component is marked in *orange*, and the attentive component is displayed in *blue-gray*

We visually inspected screen time decompositions for the Italian language group in Italy on scale ST161 and scale ST165. While the former exhibited the highest C/IER proportion of .90, the latter was part of a systematic spike shared by multiple country-by-language groups, exhibiting a C/IER proportion of .25. Screen time decompositions are displayed in Fig. 5. The observed screen time distribution for scale ST161 (Fig. 5a) did not show a bimodal shape but was heavy-tailed. The decomposition accommodated this in terms of two normal distributions with almost equal means but different variances. The smaller component had a larger variance, thus capturing the heavy tails. In the present case, the larger component yielded a somewhat smaller mean than the smaller one, resulting in identifying a counter-intuitively high (and presumably falsely labeled) C/IER proportion. The screen time decomposition of scale ST165 (Fig. 5b), in contrast, yielded comparably well-separated components. Although it is not possible to determine whether the component with the lower means indeed goes back to C/IER, this poses a plausible explanation for the pattern obtained from the screen time decomposition.

#### RO2: Initial validity checks

Overall, conducted initial validity checks were in line with our expectations and theoretical considerations on the occurrence of C/IER.

##### H1: Relating C/IER to scale characteristics

We observed no PSRF values above 1.05 in the Bayesian hierarchical Beta regression conducted to investigate the relationship between scale characteristics presumably imposing a higher cognitive burden on respondents and the occurrence of C/IER. As evidenced in Table 2, screen position and average text length were positively related to the proportion of C/IER. For the number of items and the number of response options, regression coefficients were not credibly different from zero. At the same time, there was considerable variation in baseline C/IER proportions across country-by-language groups.

**Table 2:** Results for the Bayesian hierarchical Beta regression

Fixed effects	Estimate	95% CI
Intercept	-4.196	[-4.414; -3.976]
Screen position	0.021	[0.019; 0.022]
Number of items	0.006	[-0.009; 0.022]
Number of options	0.029	[-0.003; 0.058]
Average length	0.016	[0.010; 0.023]
Random effects	Estimate	95% CI
Intercept (SD)	0.438	[0.365; 0.528]
Precision parameter	24.562	[23.269; 25.874]

SD: standard deviation; CI: credibility interval. Average length gives the average number of words per item in the English source version of the background questionnaire

Inserting the regression coefficients displayed in Table 2 into Eq. 9 yields an expected mean C/IER proportion of .03 for a scale administered at the first screen position considered (21) when all other characteristics held at their median values, i.e., 4.5 items with four response options and an average item length of ten words. Administering the same scale at the latest screen position considered (80) raises the expected mean C/IER proportion to .10.

##### H2: Investigating agreement between item omissions and C/IER behavior

**Fig. 6:**
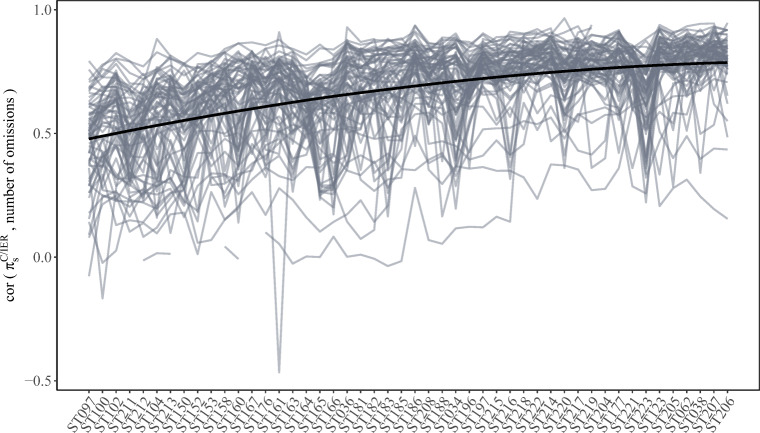
Country-by-language-group-level correlations between C/IER probabilities and the number of item omissions across the considered scales. A loess regression line is superimposed

Across all scales, we observed high correlations between C/IER probabilities and the number of item omissions per scale (average correlation across all scales: .67; see Fig. 6). Furthermore, the correlations between C/IER probabilities and the number of item omissions per scale tended to increase with later scale positions; the lowest average correlation of .46 was observed for scale ST211 (administered at position 24) and the highest of .82 for scale ST207 (administered at position 79). This may indicate that C/IER and omission behavior tended to increasingly coincide with later scale positions. The average C/IER probability for respondents leaving a scale completely unanswered was .61. At the same time, C/IER did not solely result in omissions. This becomes, for instance, evident in the fact that even for times associated with complete response patterns without any omissions, the average C/IER probability was still .02, and as high as .08 for response patterns containing both observed responses and item omissions.

##### H3: Investigating agreement with timing-based indicators

**Fig. 7:**
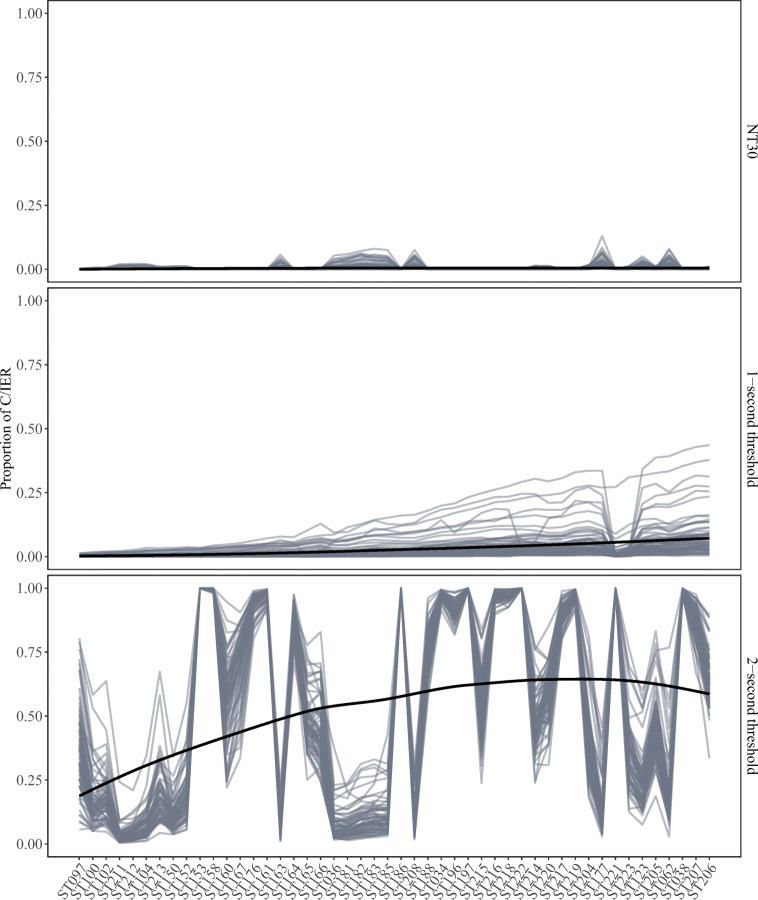
Country-by-language-group-level trajectories of C/IER proportions across the considered scales for different threshold settings. Loess regression lines are superimposed

Figure 7 displays country-by-language-group-level trajectories of C/IER proportions implied by the three investigated indicator-based procedures. Results vastly differed for different threshold settings. The 2-s threshold yielded a grand mean C/IER proportion of as high as .53 and exhibited strong variation in implied C/IER proportions across scales. For 13 scales, C/IER proportions were above .95, and for three scales (ST153, ST186, and ST221) all respondents were classified as inattentive. With .03, the 1-s threshold yielded the grand mean C/IER proportion closest to the .06 obtained from the proposed approach. The 1-s threshold yielded C/IER trajectories that may be considered most face valid, with steady increases in C/IER proportions for some country-by-language groups,^4^ and constantly low C/IER proportions for others. NT30 tended to be extremely conservative, classifying only 0.04% of respondent-by-scale interactions as inattentive. On 17 scales, NT30 yielded C/IER proportions of 0 for all country-by-language groups.

C/IER probabilities exhibited positive correlations with all timing-based indicators. Nevertheless, correlations strongly varied in size. The 2-s threshold showed the lowest degree of agreement (average correlation across all scales: .29), followed by NT30 (.38). The 1-s threshold tended to show strong agreement with C/IER probabilities (.75).

##### H4: Investigating rank-order consistency in C/IER behavior

**Fig. 8:**
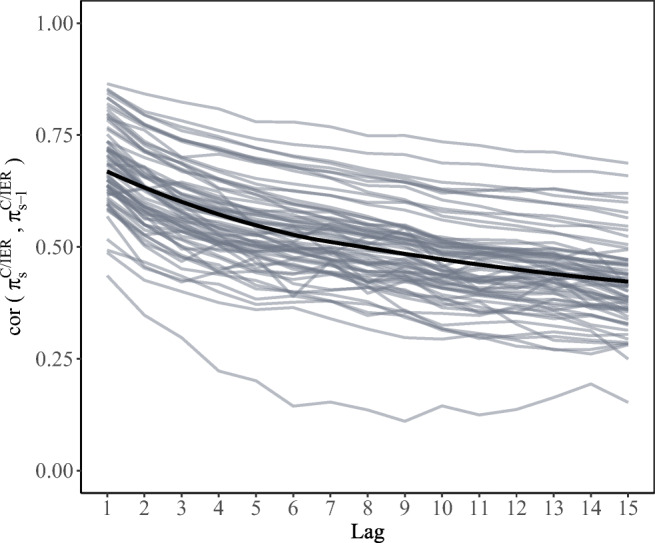
Country-by-language-group-level lagged correlations between posterior C/IER class probabilities averaged across the considered scales. A loess regression line is superimposed

Lagged correlations between individual posterior C/IER class probabilities of the considered scales, given in Fig. 8, indicated considerable rank-order stability in C/IER behavior. In line with what would be expected, lagged correlations decreased with increasing distance between the scales. While country-by-language groups differed in the level of lagged correlations—ranging, for instance, from .44 to .88 for a lag of 1—the patterns of decrease in lagged correlations were very similar across country-by-language groups.^5^

#### RO3: Investigating the impact of C/IER adjustments

For the majority of the scales considered in the present application, we found only minor differences in country-by-language group means and variances. Across all scales, the median of the scale-specific median absolute difference between unadjusted and adjusted standardized country-by-language group means was 0.01, with a middle 50% range of [0.01; 0.02]. The median of the scale-specific median absolute difference between unadjusted and adjusted standardized country-by-language group variances was 0.02 [0.01; 0.04].

The by far most pronounced differences were observable for scales ST223, ST165, and ST166, exhibiting median absolute differences between unadjusted and adjusted standardized country means of 0.17, 0.14, and 0.07, respectively. Median absolute differences in variances for these scales were 0.09, 0.13, and 0.05. With average C/IER probabilities of .17, .12, and .10, these scales were among the scales that were most strongly affected by C/IER. At the same time, C/IER behavior on these scales manifested itself not only in omissions but also in increased (presumably) aberrant responding. This is evidenced by average C/IER probabilities for complete response vectors of .10, .09, and .07, respectively, which were the highest across all scales.

## Discussion

We developed a scalable two-step screen-time-based weighting procedure for identifying and accounting for C/IER in large-scale survey data. In Step 1, the approach draws on mixture modeling to identify subcomponents of log screen time distributions presumably stemming from C/IER. In Step 2, the analysis model of choice is applied to item response data, with respondents’ posterior class probabilities being employed to downweigh response patterns according to their probability of stemming from C/IER.

The approach combines the advantages of traditional indicator-based procedures and sophisticated model-based approaches. As traditional indicator-based procedures, the approach is scalable and easy to implement, and can, as such, feasibly be integrated with common analysis workflows for large-scale survey data. It provides researchers with great flexibility in analyzing item response data with the method of their choosing, as attentiveness weights can be integrated with any type of analysis model that allows for the inclusion of sampling weights. The approach is based on screen times, which can easily be recorded on customary platforms for administering computer-based surveys, and, therefore, does not require sophisticated data pre-processing (in contrast to retrieving item-level response times from survey data as in Kroehne & Goldhammer, 2018). As sophisticated model-based approaches, the approach does not require threshold settings, allows considering the uncertainty in C/IER identification, and is agnostic towards the specific types of C/IE response patterns in the data at hand. Note, however, that as timing-based indicators, the proposed approach achieves avoiding assumptions on C/IE response patterns by imposing strong assumptions on screen time distributions.

One way to integrate the proposed approach with the analysis of survey data is to employ it for conducting sensitivity checks, investigating the robustness of results when responses that may go back to C/IER are given less weight in the analyses. Such analyses may support researchers in deciding whether or not their results are likely to be distorted by C/IER behavior of respondents (see DeSimone et al., 2018; Woods, 2006; Schmitt & Stuits, 1985; Huang et al., 2012; Kam & Meyer, 2015, for examples of biases induced by C/IER).

We illustrated the approach on a sample of more than 400,000 respondents being administered 48 scales of the PISA 2018 BQ. Conducting initial validity checks, we found a) C/IER behavior across the questionnaire to be related to scale characteristics that can be assumed to impose a higher cognitive burden on respondents, i.e., position and text length, b) convergence among C/IER probabilities and observed omission behavior, c) C/IER probabilities to exhibit strong agreement with a more heuristic 1-s-threshold C/IER indicator, and d) high rank-order consistency of individuals’ C/IER behavior across scales. These findings are in line with our expectations and, therefore, taken as initial supporting validity evidence for the proposed approach.

On most scales of the considered PISA 2018 BQ data set, C/IER tended to strongly coincide with omission behavior, such that C/IER adjustments did not heavily alter scale group means and variances. We observed pronounced impacts of C/IER adjustments on the IRT analysis results only for scales that were strongly affected by C/IER (i.e., C/IER proportions above .10) and on which C/IER respondents not only engaged in omission behavior but also to a larger extent provided aberrant responses, as indicated by increased average C/IER probabilities for respondents with complete response vectors.

In the empirical application, we further found different thresholds for creating timing-based indicators to yield vastly different results. NT30, which is an established procedure considered as liberal in the context of cognitive assessments (Wise & Kuhfeld, 2021), was highly conservative when applied for the detection of C/IER, identifying no C/IER in more than a third of the considered scales. The customary 2-s threshold, in contrast, was too high for the PISA 2018 BQ data, almost coinciding with the average time per item and sometimes classifying all respondent-by-scale interactions as inattentive. This illustrates once more that thresholds are not globally applicable and, therefore, need to be employed with caution. Although halving the 2-s threshold to 1 s yielded results that seemed face valid, it is not possible to determine whether this is the most “optimal” threshold (0.5 or 1.25 s may have been an even better choice), and any type of threshold setting will ultimately remain arbitrary. The proposed approach, in contrast, identifies C/IER by assuming that log screen times decompose into multiple subcomponents out of which the component with the lowest mean is assumed to go back to C/IER. As such, it does not require threshold settings and can—in the case that the C/IER component exhibits large dispersion—also yield higher C/IER probabilities for extremely long times spent on screen (but see Yildirim-Erbasli & Bulut, 2021, for threshold settings accommodating long times).

In the application and evaluation of the proposed approach, we focused on LSA BQ data, as we see these as important use cases. We could show that the approach can easily be implemented without strong increase in computational burden. At the same time, it allows for closely mimicking key elements of operational practice, such as the inclusion of sampling weights, and provides researchers with great flexibility in employing the analysis model of their choosing for attentive item responses. We also point out that the approach can also be applied to other types of surveys that provide timing data on the screen level. Note that sample size requirements are determined by various factors, including the complexity of the chosen model for analyzing response data (i.e., whether a complex IRT model is employed or whether attentiveness weights are used to calculate weighted subgroup mean sum scores) and whether or not mixture components are well separated. As a rule of thumb for univariate Gaussian mixture models, Srebro, Shakhnarovich, and Roweis (2006) recommended sample sizes of at least 8/*d*^4^, where *d* gives the smallest standardized mean difference between any two components. For instance, when screen time distributions associated with attentive and C/IER behavior are assumed to have medium separability, say *d* = 0.5, the minimal sample size required for trustworthy mixture decomposition of screen times would be 8/0.5^4^ = 128. For small separability of *d* = 0.2, according to the rule of thumb by Srebro et al., (2006), a sample size of at least 8/0.2^4^ = 5000 would be required.

On a more general note, we caution researchers to take the potential occurrence of C/IER in sample size planning into account. Downweighing or eliminating presumed C/IE response patterns effectively lowers the sample size of the data at hand, as not all observed data points (fully) contribute to parameter estimation.

### Limitations and future directions

Avoiding assumptions on C/IER response patterns comes at the price of strong distributional assumptions for screen times. Violations of distributional assumptions are known to distort the performance of timing-based mixture models (Molenaar, Bolsinova, & Vermunt, 2018). As such, the quality of C/IER identification may be heavily contingent on whether or not the distributional assumptions that the Gaussian mixture decomposition of log screen times entails are met (see also Bauer & Curran, 2003, for a cautionary note). In fact, in investigating selected screen time decompositions in the empirical application, we could show how violations of distributional assumptions (e.g., heavy tails) may yield counter-intuitive results and false C/IER labels. We, therefore, recommend to visually inspect decompositions that yield counter-intuitive results, such as sudden spikes or unusually high C/IER proportions, and scan for indications that these may be artifacts going back to violations of distributional assumptions. Further investigating the robustness of the proposed approach to violations of the normal assumption for C/IER screen time mixture components and developing solutions for such scenarios is, therefore, an important task for future research.

One of the main advantages of the proposed approach over model-based mixture modeling approaches to C/IER (Ulitzsch et al., 2021; Ulitzsch et al., 2022) lies in its scalability. Compared to more sophisticated model-based procedures, however, the proposed approach determines C/IER probabilities on each scale separately and leaves information on C/IER behavior on preceding scales unconsidered. As C/IER behavior can be assumed to generalize across scales to some extent (Bowling et al., 2016), extensions that leverage information on C/IER behavior on preceding scales are a promising topic for future research. A potential starting point may be to attempt incorporating C/IER probabilities of preceding scales as prior information in the decomposition of screen times.

Likewise, the approach neglects the information contained in response patterns. Future research may develop procedures that, in analogy to the threshold setting approach by Soland et al., (2019), additionally leverage information on aberrances in response patterns in the decomposition of screen times, e.g., by regressing class membership on a set of response-pattern-based indicators that are sensitive to different aspects of C/IER, such as the long string index and Mahalanobis distance, or by considering response-pattern-based indicators jointly with screen times in multivariate Gaussian mixture models.

As is common for filtering, mixture, and weighting procedures for accounting for C/IER in non-cognitive assessments (Hong and Cheng, 2019; van Laar & Braeken, 2022; Arias et al., 2020; Meade & Craig, 2012; but see Ulitzsch et al., 2021; Ulitzsch et al., 2022, for exceptions) and rapid guessing in cognitive assessments (Wang & Xu, 2015; Wise & DeMars, 2006; Pokropek, 2016; but see Ulitzsch et al., 2020; Liu, Li, Liu, & Luo, 2019; Nagy & Ulitzsch, 2021, for exceptions), the proposed approach assumes the mechanism underlying C/IER to be unrelated to the traits to be measured.^6^ C/IER behavior, however, has repeatedly been found to be related to various respondent characteristics such as demographic variables (e.g., education, gender, or geographic location; Kim, Dykema, Stevenson, Black, & Moberg, 2018; McKay et al., 2018), personality traits (e.g., the Big Five, self-esteem, or need for structure; Maniaci & Rogge, 2014; Bowling et al., 2016; Huang et al., 2015; Nichols & Edlund, 2020; McKay et al., 2018), verbal ability (Knowles, Cook, & Neville, 1989), or the interest in the survey content (Brower, 2018; see Soland et al., 2019, for a discussion of correlates of rapid guessing behavior in cognitive assessments), rendering this assumption likely to be violated in practice. Therefore, a pertinent topic for future research is to expand the proposed procedure to allow for attentiveness weights to be correlated to the traits to be measured.

Even though our initial validation checks provided first supporting validity evidence for the proposed approach, further studies that probe the validity of the proposed approach are needed. These may investigate agreement with other behavioral or self-report measures of C/IER, relationships with known antecedents and correlates of C/IER (as in Ulitzsch, Penk, von Davier, & Pohl, 2021) or investigate validity by means of experimental studies, evoking or curbing C/IER behavior by varying instructions or incentives, and investigating whether the approach is sensitive to differences in C/IER among experimental groups (as in Niessen et al., 2016; Bowling et al., 2021).

Further validation studies may also compare different approaches to identifying C/IER components in the screen time decompositions. We proposed to label only a single component—the component with the lowest mean—as due to C/IER. This labeling, however, is not without alternatives. There sometimes may be more than one component comprising extremely short screen times. For some decompositions, it may be reasonable to label components with extremely high means as due to C/IER, especially when respondents are administered the questionnaire on their own devices and may get distracted by other browser tabs or applications, thus requiring a long time to provide inattentive responses (see also Yildirim-Erbasli & Bulut, 2021). Further, it may be that some decompositions do not contain any component stemming from C/IER. It is an important task for future research to develop more flexible, sophisticated, and possibly more valid procedures to labeling C/IER components.

We validated and applied the approach using scales that are reflective of non-cognitive constructs, measuring personality, attitudes, interests, beliefs, and behavior. It remains to be investigated how well the approach works for factual questions (e.g., self-reports of demographics such as age or gender), single-item scales, or items with an open-response format. Positive evaluations of the proposed approached for such types of items would considerably broaden its scope of application, rendering it a useful tool for fields outside of psychology and educational science where IRT is rarely employed and single-item scales are more common (e.g., market and consumer research).

The proposed approach adjusts for C/IER at the person level by deriving person attentiveness weights from screen times. In the empirical application, we could analyze C/IER trajectories across the PISA 2018 BQ because we analyzed each scale separately (as is done in operational practice) and each scale was presented on one screen. A pertinent topic for future research is the development of scalable approaches that extend the presented one to the joint analysis of items presented on different screens, while taking into account that C/IER may vary across screens. This may be especially important when researchers want to adjust estimates of relationships among different scales (presented on separate screens) for C/IER.

## Footnotes

1 Note that this does not entail that only short response times are treated as indicators of C/IER. In fact, Nagy and Ulitzsch (2021) pointed out that when the distribution of C/IE response times displays large variability, which is often found to be the case in applications of related models, it may also cover aberrantly large response times that exceed values typically expected under attentive responding.

2 As with a median of 4.5 items the analyzed PISA 2018 BQ scales were commonly very short, we deemed response-pattern-based indicators such as the long string index as uninformative and, therefore, focused on omissions and timing data.

3 A detailed description of the design, sampling procedure, and administration can be found in OECD (2020). Data are publicly available at https://www.oecd.org/pisa/data/2018database/. The English source version of the BQ is available at https://www.oecd.org/pisa/data/2018database/CY7_201710_QST_MS_STQ_NoNotes_final.pdf.

4 Interestingly, the only exception to this steady increase were scales ST221 and ST223. For these scales, C/IER proportions abruptly dropped, contradicting results obtained from the proposed approach. Recall that for this pair of scales, the proposed approach yielded a spike in C/IER proportions.

5 The only exception was the English language group in Malaysia, for which lagged correlations were somewhat lower than in the remaining country-by-language groups.

6 Note that this limitation holds regardless of whether approaches identify C/IER (or rapid guessing) on the respondent or item-by-respondent level (see Ulitzsch et al., 2020; Pokropek, 2016; Rios, Guo, Mao, & Liu, 2017, for further discussions and illustrations).
